# Carbon Nanotube-Quicklime Nanocomposites Prepared Using a Nickel Catalyst Supported on Calcium Oxide Derived from Carbonate Stones

**DOI:** 10.3390/nano9091239

**Published:** 2019-08-31

**Authors:** Ruzanna Ibrahim, Mohd Zobir Hussein, Nor Azah Yusof, Fatimah Abu Bakar

**Affiliations:** 1 Materials Synthesis and Characterization Laboratory (MSCL), Institute of Advanced Technology (ITMA), Universiti Putra Malaysia, Serdang 43400, Selangor, Malaysia; 2 Functional Devices Laboratory (FDL), Institute of Advanced Technology (ITMA), Universiti Putra Malaysia, Serdang 43400, Selangor, Malaysia; 3 Department of Food Science, Faculty of Food Science and Technology, Universiti Putra Malaysia, Serdang 43400, Selangor, Malaysia

**Keywords:** calcium oxide, carbon nanotubes, chemical vapor deposition, nanocomposites, screen printed electrodes

## Abstract

Carbon nanotube-quicklime nanocomposites (CQNs) have been synthesized via the chemical vapor deposition (CVD) of n-hexane using a nickel metal catalyst supported on calcined carbonate stones at temperatures of 600–900 °C. The use of a Ni/CaO(10 wt%) catalyst required temperatures of at least 700 °C to obtain XRD peaks attributable to carbon nanotubes (CNTs). The CQNs prepared using a Ni/CaO catalyst of various Ni contents showed varying diameters and the remaining catalyst metal particles could still be observed in the samples. Thermogravimetric analysis of the CQNs showed that there were two major weight losses due to the amorphous carbon decomposition (300–400 °C) and oxidation of CNTs (400–600 °C). Raman spectroscopy results showed that the CQNs with the highest graphitization were synthesized using Ni/CaO (10 wt%) at 800 °C with an I_G_/I_D_ ratio of 1.30. The cyclic voltammetry (CV) of screen-printed carbon electrodes (SPCEs) modified with the CQNs showed that the performance of nanocomposite-modified SPCEs were better than bare SPCEs. When compared to carboxylated multi-walled carbon nanotubes or MWNT–COOH-modified SPCEs, the CQNs synthesized using Ni/CaO (10 wt%) at 800 °C gave higher CV peak currents and comparable electron transfer, making it a good alternative for screen-printed electrode modification.

## 1. Introduction

Carbon nanotubes (CNTs) are a type of nanomaterial that has been intensively investigated after it was first reported by Iijima in 1991 [1]. The attractive features of CNTs for a myriad of applications are superior mechanical strength, chemical stability, high electrical conductivity, and biocompatibility [2]. Production of CNTs has been achieved through three main techniques, namely, arc discharge, laser ablation, and chemical vapor deposition (CVD) [3]. The CVD technique is able to achieve a high yield of CNTs at relatively low temperature compared to the other two methods [4].

In CVD techniques, hydrocarbon gases, for example acetylene are usually used as the carbon feedstock. Alternatively, a liquid carbon source such as hexane can also be used. Liquid carbon sources such as hexane and alcohols, which were introduced by Maruyama et al. [5], are attractive for use in CNT production via CVD, as it can eliminate usage of large containers (gas tanks) and valves. In addition, a CVD system which utilizes liquid carbon source is versatile as it can be used with many types of liquid carbon precursors [6]. Support materials with high surface areas, such as alumina (Al_2_O_3_) and silicon dioxide (SiO_2_), are conventionally used to support the metal catalysts in CNT production [7]. In this study, we describe the use of a support material obtained from a natural source, which is local carbonate stone. The carbonate stones are a good source of CaO (after calcination) which can act as the catalyst support for using Ni metal catalysts to synthesize CQNs via chemical vapor deposition of hexane. Carbonate stones were chosen as the CaO source since they are natural, easily available, and are more economical for preparing catalyst support for CNT synthesis via CVD.

Calcium oxide is commonly known as lime, burnt lime or quicklime. Calcium oxide is usually used in the manufacture of various products such as glass, paper, and steel. It is also used in building and construction materials, in agriculture as fertilizer, in the food industry, in flue gas purification, and also in water/sewage treatment. Recent works investigated the use of CaO in catalysis and as gas adsorbent at high temperature. For instance, Buasri et al. [8] studied the application of CaO and MgO from dolomite rock as a catalyst for the synthesis of biodiesel. In other works, CaO stabilized using zirconium(ZrO_2_) has been used to construct oxygen sensors for metallurgical melts. This is because CaO-doped zirconium oxide exhibits increased toughness compared to other metal oxides [9,10,11]. A previous study also explored the use of a chitosan-CaO nanoparticle-MWNT modified-gold electrode for the determination of the Tartrazine dye in food [12,13]. Calcium oxide or quicklime is a robust material that can withstand high temperatures making it versatile, expanding its potential applications.

Previous works using CaO as catalyst support for CNT synthesis are fairly rare and most previous studies used calcium carbonate (CaCO_3_) which also produces CaO in the final product [14,15,16,17]. Additionally, CNTs previously synthesized via CVD using CaCO_3_ as catalyst support and acetylene as a carbon source claimed to achieve CNTs with no formation of any amorphous carbon [15], and it was also reported that the amorphous carbon yield was insignificant [16]. Among the motivations for using CaO as the catalyst support compared to other support materials is that CaO can easily be removed if purification of the CNTs is required using a high concentration of nitric or sulphuric acid [18]. Additionally, CaO also adds more thermal stability to the final nanocomposite as it can withstand high temperatures.

In addition, using CaO support also allows the production of CNT-quicklime nanocomposites which may be suitable for CNT-based advanced building materials, as CaO is a common component of such materials. Previous findings indicate that the addition of CNTs to various substances such as cement mixtures was able to improve the strength of the cement matrix [18,19]. The incorporation of fibers such as CNTs into cementitious materials such as concrete and bone cement was done to overcome limitations, such as low tensile strength, low strain capacity, and also to achieve possible reduction and eventual elimination of microcracks [20,21]. Sobolkina et al. [22] has found that using a CNT-to-surfactant ratio of 1:1–1:1.5 and a sonication time of 120 min yielded the best CNT dispersion, as this is usually the limitation in this type of CNT application.

Apart from the use as electrode materials for sensors and advanced building materials, CNT-quicklime nanocomposites are also useful for a number of other applications where CNT and CaO play essential roles. For instance, CNT-quicklime nanocomposites can be used as catalyst in biodiesel production since CaO is a suitable catalyst for biodiesel production. The presence of CNTs could increase the surface area of the catalysts, therefore it is a promising candidate to be used as a catalyst for biodiesel production [23,24,25]. For a similar application, other carbon materials such as graphite oxide have also been combined with CaO for biodiesel production [26]. Another area of which CNT-quicklime nanocomposites may be used is as electrode materials for supercapacitors. This is because the capacitive performance of CNTs have improved with the addition of various metal oxides. Therefore, a combination of CaO and CNT in CNT-quicklime nanocomposites may also be a suitable material for electrochemical supercapacitor applications [27]. In addition, CaO has been applied in thermochemical heat storage for solar power plants and CNTs were used as solar cell electrode materials; therefore, it is also possible to use the synthesized CNT-quicklime nanocomposites for these purposes [28,29]. Thus, it is worthwhile to investigate the production and properties of CNT-quicklime nanocomposites as it has potential use in many applications as mentioned above.

In this work, CNT-quicklime nanocomposites (CQNs) were synthesized using hexane CVD, and the CaO-supported metal catalyst used was nickel. The aim of this work was to synthesize CQNs from CaO-supported Ni catalysts and to characterize the CQN product obtained. The CaO in this work was obtained from calcined carbonate stones. This paper also included a study on the effect of different CVD temperatures and catalyst compositions on the properties of the synthesized CQNs. Furthermore, the possible usage of CNT-quicklime nanocomposites (CQNs) as electrode modifiers for use in electrochemical detection were also explored.

## 2. Materials and Methods

All the chemicals used in this study were used as received, without further purification. The metal salt used in catalyst preparation was nickel (II) nitrate hexahydrate (Ni(NO_3_)_2_∙6H_2_O) (99%) which was purchased from Acros Organics (New Jersey, USA) and *n*-hexane which was obtained from Merck (Darmstadt, Germany). Potassium hexacyanoferrate (III) (K_3_[Fe(CN)_6_])(99%) and potassium chloride (KCl) (99%) was from Nacalai Tesque (Kyoto, Japan). Carboxylated multi-walled carbon nanotubes (MWNT–COOH) (95%) was from NovaScientific Resources (M) Sdn. Bhd, Selangor, Malaysia. The carbonate stones were obtained locally and were washed and dried prior to treatment. Screen printed carbon electrodes (SPCEs) were obtained from Scrint Technology (M) Sdn. Bhd, Kedah, Malaysia. Deionized water was used throughout the experiment.

### 2.1. Catalyst Preparation

The catalyst support, CaO, was obtained by calcining crushed carbonate stones at 1000 °C for 5 h. The calcined stones were then finely ground into fine powder and sieved (90 μm) prior to the catalyst preparation. Supported metal catalysts were prepared similar to Reference [15]. Briefly, an appropriate amount (5, 10, 15, and 20 wt%) of Ni nitrate salts were dissolved in 40 mL distilled water. Then, the metal catalysts were impregnated onto the CaO support by direct addition of CaO into the metal salt solution. The mixture was continuously stirred and heated (70 ± 10 °C) to evaporate the solvent. The resulting product was then dried in an oven at 70 ± 10 °C overnight and was kept in a sample bottle for further use and characterization. All the catalysts were sieved using a 90 μm sieve before being subsequently used in the CVD experiments.

### 2.2. Synthesis of Carbon Nanotube Quicklime Nanocomposites

The carbon nanotubes-quicklime nanocomposite (CQNs) synthesis by hexane decomposition which was carried out in a horizontal furnace at a specified temperature. Approximately 1 ± 0.005 g of the prepared catalyst was placed in an alumina boat and inserted into a quartz tube (OD 25 mm × ID 20 mm × 1000 mm) before placing the tube inside the horizontal furnace. The tubes were then connected to the gas pipe and then a nitrogen flow (100 mL/min) was introduced into the reaction chamber and the system was heated to a specified temperature. When the desired temperature was reached, the hexane flow was introduced by allowing the nitrogen carrier gas to flow over the hexane at atmospheric pressure [30]. At the end of the experiment, the furnace was switched off and left to cool down to room temperature under nitrogen flow. The final product was collected from the alumina boat and was ground and stored in a sample bottle for further use. The CQNs that were synthesized using hexane and nickel catalysts are denoted as HNi followed by numbers that indicate the metal percentage of the catalyst (e.g., HNi10 is nickel catalysts with 10 wt% nickel). Additionally, the samples were also labelled with the corresponding temperature used in the synthesis procedure.

### 2.3. Material Characterizations

Powder X-ray diffraction (XRD) of the crystalline phase identification was carried out using a Shimadzu diffractometer (XRD6000) (Shimadzu, Tokyo, Japan) with Cu Kα (30 kV) radiation. The XRD patterns were recorded in the 2θ range of 10–80° with a scanning speed of 4°/min. Field emission scanning electron microscopy (FESEM) images were recorded with a Nova Nanosem 30 series microscope (FEI Company, Oregon, USA) using 5 kV. Internal morphologies of the samples were obtained via transmission electron microscopy (TEM) using the H-7100 microscope from Hitachi (Tokyo, Japan). Prior to the TEM measurements, the samples were dispersed in ethanol by sonication for 15 min before it was dropped on the copper grid. Textural properties of the samples were determined using nitrogen gas adsorption–desorption techniques with ASAP2000 analyzer, (Micromeritics, Georgia, GA, USA) with degassing was done at 105 °C overnight. The thermal decomposition of the product was investigated using a thermogravimetric analyzer, (TA Instruments, Delaware, DE, USA). The temperature range used for the thermal analysis was from room temperature to 1000 °C with a heating rate of 5 °C/min. Raman spectra of the CQNs were obtained using a Raman spectrometer (WITec, Ulm, Germany) with a 532 nm laser.

### 2.4. Modification of Screen-Printed Carbon Electrodes and Electrochemical Measurement

Screen-printed carbon electrodes (SPCEs) were obtained from Scrint Technology Sdn. Bhd, Malaysia. The SPCEs consisted of a carbon working electrode, an Ag/AgCl_2_ reference electrode, and a carbon counter electrode. The SPCE structure was made of plastic and all SPCEs were disposed after each use.

#### 2.4.1. Modification of Screen-Printed Electrodes Using Nanomaterials

The electrochemical behavior of the nanocomposites was studied using modified screen-printed carbon electrodes (SPCEs). Prior to the SPCEs modification, a dispersion of the modifier nanomaterial was prepared in ethanol via sonication for 30 min. Subsequently a desired volume of dispersion was dropped onto the working electrode of the SPCEs using a micropipette. The material was left to dry on the SPCEs overnight at room temperature.

#### 2.4.2. Cyclic Voltammetry Studies of the Modified SPCE

The electrochemical characterization of the modified SPCEs was conducted via cyclic voltammetry (CV) using a computer controlled Dropsens 8400 electrochemical system (Metroohm Co., Herisau, Switzerland). The potential was varied between −0.6 V to 0.6 V and the scanning speed used was 100 mV/s. The CV analysis was conducted for the redox reaction of the ferrocyanide/ferricyanide redox couple using a 0.01 M K_3_[Fe(CN)_6_] and 0.1 M KCl solution as the electrolyte. A new electrolyte solution was used for each batch of analysis to avoid any contamination. All CV analysis was conducted in unstirred solution at room temperature. The analysis was done in triplicates.

## 3. Results and Discussions

### 3.1. X-Ray Diffraction Results

**Figure 1:**
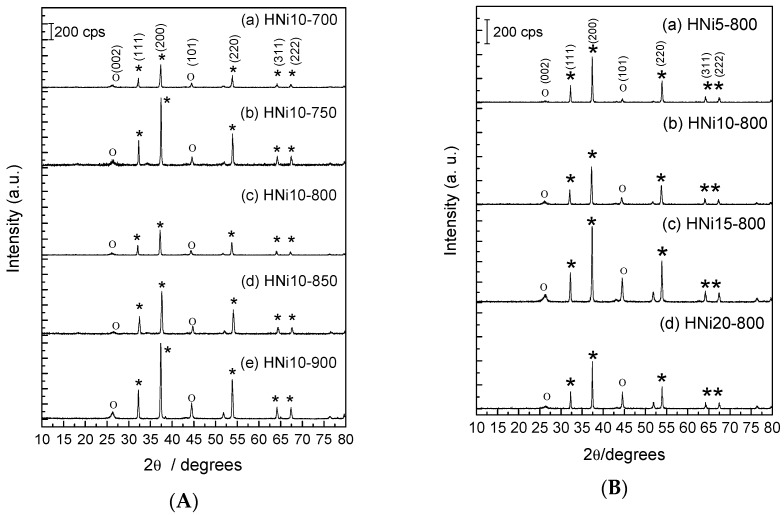
(**A**) XRD patterns of carbon nanotubes-quicklime nanocomposites, CQNs prepared using a Ni/CaO catalyst (10 wt%) at (a) 700 °C, (b) 750 °C, (c) 800 °C, (d) 850 °C, and (e) 900 °C ((o) CNT, (*) catalysts and support); (**B**) XRD patterns of CQNs carbonized at 800 °C prepared using a Ni/CaO catalyst with Ni composition of (a) 5 wt%, (b) 10 wt%, (c) 15 wt%, and (d) 20 wt% ((o) CNT, (*) catalysts and support).

The CQNs synthesis was carried out at 600–900 °C. Nickel catalyst at metal percentage of 10 wt% was used to determine the suitable reaction temperature. The XRD patterns for the CVD product obtained from Ni/CaO (10 wt%) and HNi10 at various heating temperatures are shown in Figure 1a. Meanwhile Figure 1b illustrates the XRD patterns obtained when CQNs were synthesized using different compositions of Ni/CaO catalysts at 800 °C.

The XRD patterns for CQNs synthesized using Ni/CaO (10 wt%) at 700 to 900 °C is shown in Figure 1a, and the possible presence of CNTs in the sample was expected to be indicated with a peak at 2θ = 26°. The peak for the (002) reflection, which is usually observed at 26.5°, and another peak at 44° can be attributed to graphite, indicating the (101) reflection (graphite, JCPDS: 00-001-0640) [31,32]. Reflections at 32°, 38°, 54°, 64°, and 67° can be attributed to catalysts and catalyst supports (CaO, JCPDS: 01-074-1226) reflections of (111), (200), (220), (311), and (222), respectively. Only temperatures as low as 700 °C can be used to synthesize CQNs with the Ni/CaO catalysts as indicated by the XRD results. A similar result was obtained with acetylene as the carbon source and zeolite supported metal catalysts for CNTs synthesis via CVD method [33]. As shown in Figure 1a, the intensity of the XRD reflections increased with the increase of reaction temperature, indicating an increase in the amount of CNTs in the CQNs or it may also suggest a reduction in the formation of amorphous carbon. The XRD patterns for the samples in Figure 1a also showed that at 900 °C, the 10 wt% Ni/CaO catalyst gave the reflections with the highest intensities. However, at this temperature, there were also a number of additional peaks in the XRD patterns. These extra patterns may be attributable to the formation of amorphous carbon, as was previously reported, the concomitant increase of amorphous carbon formation outside the temperature range of 800–825 °C [34].

In order to study the effect of catalyst composition, the composition of the Ni/CaO catalysts was varied at 5, 10, 15, and 20 wt% nickel, while the synthesis temperature was maintained at 800 °C, while the holding time was kept constant at 30 min. It can be observed from Figure 1b that as the nickel content was increased up to 15 wt%, the XRD reflection intensity was also increased, indicating more CNTs were produced in the samples. However, at 20 wt% the intensity for the XRD peaks decreased slightly. Figure 1b also displays the presence of characteristic CNT peaks when the catalyst composition was varied. The highest peak intensity of (002) reflection at 26° was exhibited by HNi15-800. Asymmetric XRD peaks present in the XRD spectra indicates the presence of various types of crystalline features (i.e., stacked graphene sheets/CNTs) [31].

### 3.2. Raman Spectroscopy

**Figure 2:**
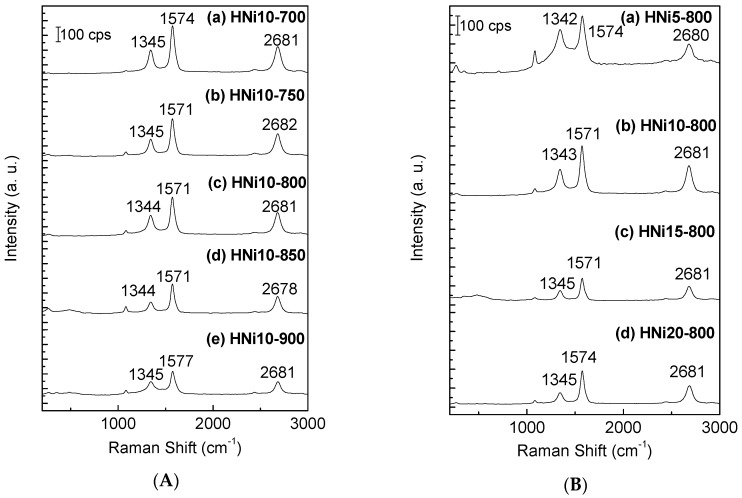
(**A**) Raman Spectra of (a) HNi10-700, (b) HNi10-750, (c) HNi10-800, (d) HNi10-850, (e) HNi10-900; (**B**) Raman Spectra of (a) HNi5-800, (b) HNi10-800, (c) HNi15-800, (d) HNi20-800.

Raman spectroscopy was used to determine the quality of the CNTs present in the CQNs. The main features in Raman spectra for CNTs are the G- and the D-lines. The G-line (usually around 1350 cm^−1^) refers to the characteristic feature of the graphitic layers (single-crystal graphite) and corresponds to the tangential vibrations of the carbon atoms. The D-line (usually around 1580 cm^−1^) can indicate a defective graphitic structure [35] or disordered carbon [36]. The quality of the CNTs is given by the ratio of the peak intensities of G-line (I_G_) to D-line (I_D_). The I_G_/I_D_ value will reflect the graphitization of the CNTs. It also displays a higher degree of structural order and purity of the nanomaterials being examined [37]. In this study, the I_G_/I_D_ ratio was used to assess the quality of the CNTs in the CQNs. Figure 2a,b shows the Raman spectrum of the selected CQNs at different chemical vapour deposition temperatures and catalyst compositions. Figure 2a,b shows the I_G_/I_D_ ratio of the CQNs obtained using Ni/CaO (10 wt%) catalysts at different CVD temperatures and catalyst compositions, respectively. It can also be observed from the Raman spectra in Figure 2 that the positions of the I_D_ and I_G_ peaks were almost constant in the range of 1350 and 1580 cm^−1^, which is in agreement with previous studies.

**Figure 3:**
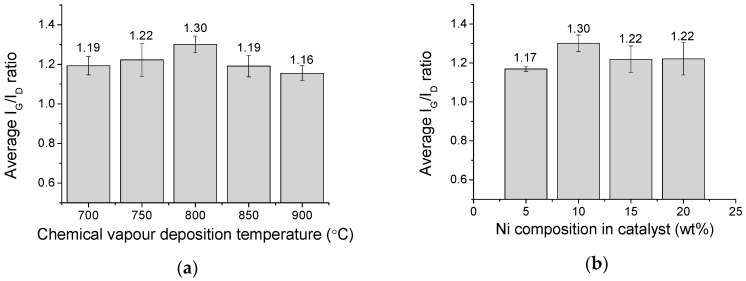
(**a**) The I_G_/I_D_ value for CQNs synthesized using Ni/CaO (10 wt%) (HNi10) at various carbonization temperatures; (**b**) the I_G_/I_D_ value for CQNs synthesized using Ni/CaO at 800 °C at different catalyst compositions.

As shown in Figure 3, Raman spectroscopy exhibited the highest I_G_/I_D_ value at 800 °C (1.30) for CQNs using Ni/CaO (10 wt%), which indicates that the CNTs in this sample possessed the highest graphitization degree compared to the CNTs synthesized using other metal compositions. Generally, the I_G_/I_D_ ratio can be observed to increase with the increment of Ni content in the catalyst. Therefore, mostly good graphitization of CNTs synthesized via CVD using CaO as a metal catalyst support are more achievable at higher metal composition in the catalyst. The presence of a band at around 200 cm^−1^ for some samples indicated that the samples obtained were multi-walled carbon nanotubes (MWNTs) with possible co-presence of SWNTs.

### 3.3. BET Surface Area and Porosity of Ni-Catalyzed CQNs

The surface area and porosity of CQN samples synthesized using Ni/CaO (10 wt%) catalysts at different carbonization temperatures were obtained using N_2_ adsorption–desorption and the results of the analysis are shown in Table 1.

As can be seen in Table 1, the surface area of the CQNs synthesized using Ni/CaO (10 wt%) decreased as the temperature of the CVD reaction increased from 700 °C to 900 °C and from 13 m^2^/g to 7 m^2^/g. This result indicates that 700 °C is the best carbonization temperature to obtain the highest BET (Brunauer-Emmett-Teller) surface area of CQNs using this type of catalyst. A similar trend was observed for the total pore volume of the samples which were 0.084, 0.078, 0.069, 0.051, and 0.063 cm^3^/g for the carbonization temperatures of 700 °C, 750 °C, 800 °C, 850 °C, and 900 °C, respectively. The lower values of BET specific surface areas for the CQNs compared to those found in the literature may be due to the presence of metal catalysts and support in the nanocomposites [38]. However, it was observed that the synthesized CQNs (using Ni/CaO (10 wt%) catalyst) all exhibited higher BET surface areas compared to the Ni/CaO (10 wt%) catalyst which was about 2 m^2^/g. The increase in BET surface area after carbonization of the catalyst could be attributed to the presence of CNTs in the nanocomposite.

Additionally, a low catalyst composition of 5 wt% is not suitable for CNTs growth due to the limited amount of metal catalyst. On the other hand, the higher metal composition of 20 wt% is also not favourable for the growth of carbon CNTs due to the generation of large catalyst particles which leads to lower surface area of the catalyst and also lower surface area of the product, since larger diameter CNTs will be formed [7,33,39]. Therefore, it is possible that for the CaO supported Ni catalyst, CNT synthesis via hexane CVD is suitable at Ni composition of 10–15 wt%.

In contrast, the pore diameter values for all the three samples increased with the increase in the CVD temperature. The catalyst composition also plays a vital part in the surface area value of the synthesized CNTs. The HNi15-800 gave the highest value for BET surface area and was closely followed by HNi10-800. The lowest and highest Ni catalyst composition, 5 wt% and 20 wt%, both gave lower values of BET specific surface area at 800 °C.

### 3.4. Field Emission Scanning Electron Microscopy

**Figure 4:**
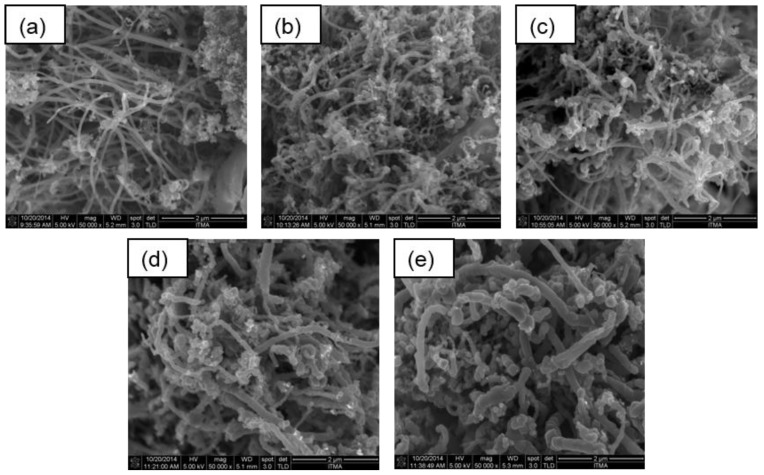
The field emission scanning electron microscopy, FESEM images of CQNs synthesized using nickel catalysts at 10 wt% synthesized at (**a**) 700 °C; (**b**) 750 °C; (**c**) 800 °C; (**d**) 850 °C; and (**e**) 900 °C. Magnification 50,000×.

**Figure 5:**
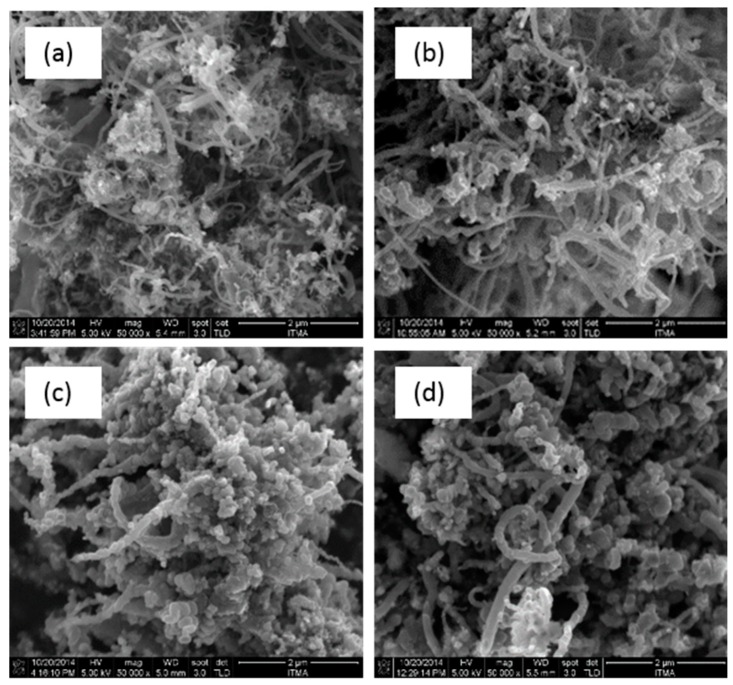
The FESEM images of CQNs synthesized using nickel catalysts at (**a**) 5 wt%; (**b**) 10 wt%; (**c**) 15 wt%; and (**d**) 20 wt% synthesized at 800 °C. Magnification 50,000×.

**Figure 6:**
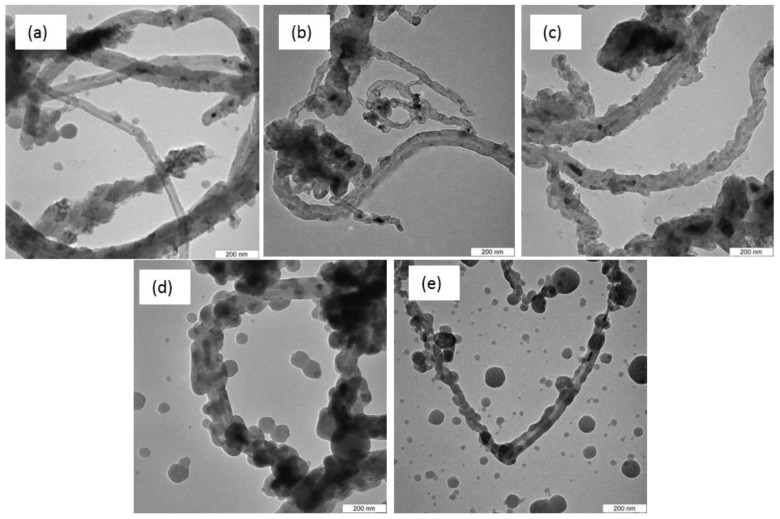
Transmission electron microscopy, TEM images of Ni-catalyzed CQNs synthesized using Ni/CaO (10 wt%) at different temperatures: (**a**) 700 °C; (**b**) 750 °C; (**c**) 800 °C; (**d**) 850 °C; and (**e**) 900 °C. Magnification at 100,000×.

The morphologies of the synthesized CQNs were investigated using field emission scanning electron microscopy (FESEM). The FESEM images are shown in Figure 4 and Figure 5. It can be seen from the FESEM images in Figure 5 and Figure 6 that carbon nanofibres are also present in the synthesized samples. All FESEM images also showed round-shaped particles that can possibly be attributed to the presence of the supported metal catalysts and other carbon nanostructures (e.g., nanospheres, nanoonions). It is also evident from the FESEM images that the carbon fibres in the nanocomposites had varying diameters.

### 3.5. Transmission Electron Microscopy

**Figure 7:**
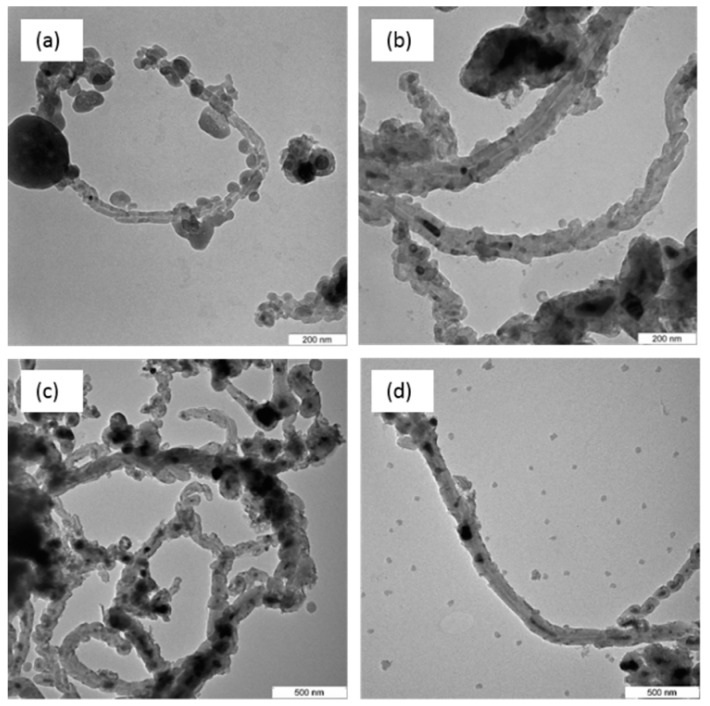
TEM images of Ni-catalyzed CQNs at 800 °C with (**a**) 5 wt; (**b**) 10 wt%; (**c**) 15 wt%; and (**d**) 20 wt% Ni. Magnification at 50,000×.

Confirmation of the presence of CNTs in the CQNs was conducted using transmission electron microscopy (TEM). The TEM images for CQNs prepared using Ni/CaO (10 wt%) at different temperatures are presented in Figure 6, while Figure 7 shows TEM image for HNi synthesized at 800 °C at various Ni percentages.

All the TEM figures show that the CQNs produced from the CVD process did possess a tubular morphology and this confirms the presence of CNTs in the nanocomposites. Catalyst particles with spherical shape and support particles can also be seen coating the outside part of the nanotubes. Additionally, as shown in Figure 6 and Figure 7, the tips of the tubes exhibited dark points which can be attributed to the incorporation of the metal catalyst into the CNTs during the growth process [34,40,41]. Some parts of the tubes are filled with the catalyst, probably due to the high-metal-to-carbon ratio in the reaction chamber [24]. Hoyos-Palacio et al. [34] attributed the encapsulation as a result of the CNT growth process and minimal interaction between the metal catalyst and its support. It can also be observed that CQN synthesized using Ni/CaO (10 wt%) gives CNTs with chain-like segments which was also observed in a previous study [38].

### 3.6. Thermogravimetric Analysis

**Figure 8:**
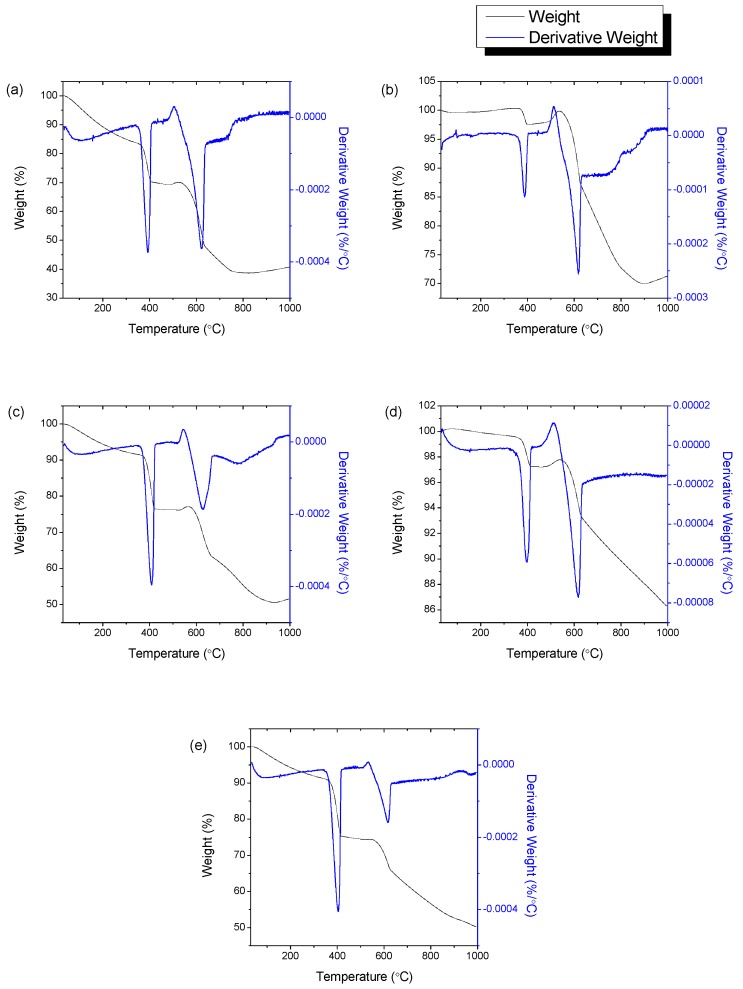
The thermogravimetric, TG and derivative thermogravimetric, DTG curves of CQNs synthesized using a Ni/CaO (10 wt%) catalyst at (**a**) 700 °C; (**b**) 750 °C; (**c**) 800 °C; (**d**) 850 °C; (**e**) 900 °C.

**Figure 9:**
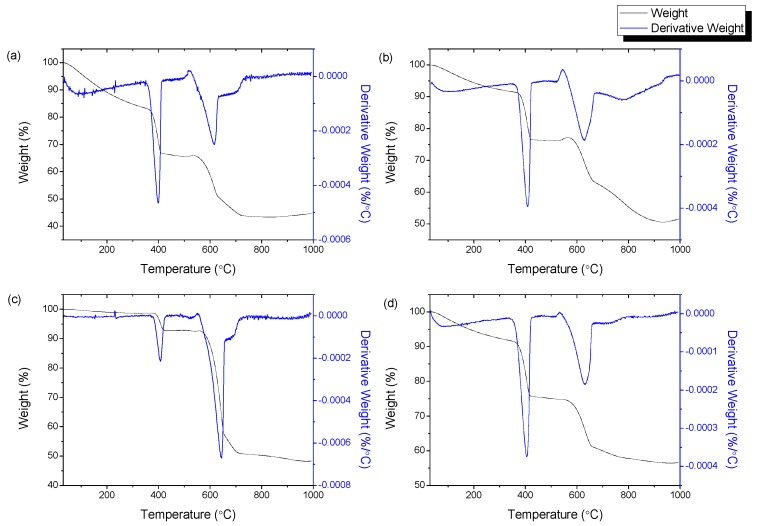
The TG and DTG curves of CQNs synthesized at chemical vapor deposition temperature of 800 °C and Ni composition of (**a**) 5 wt%; (**b**) 10 wt%; (**c**) 15 wt%; and (**d**) 20 wt%.

Thermogravimetric analyses of the CQNs were conducted to assess the thermal decomposition of the materials. The thermogravimetric (TG) and derivative thermogravimetric (DTG) curves of the CQNs produced from Ni/CaO (10 wt%) synthesized at different chemical vapour deposition temperatures are exhibited in Figure 8, meanwhile Figure 9 shows the TG and DTG curves of HNi at different Ni catalyst compositions. Table 2 presents the corresponding weight losses at the recorded temperature range determined by thermogravimetric analysis.

The TGA indicated that the highest weight loss, due to the fact of amorphous carbon combustion (300–400 °C), was observed for CQNs produced from Ni/CaO 10 wt% at 800 °C, HNi10-800 which was 18.35 wt% as shown in Table 2. The second weight loss at 400–600 °C, HNi10-900 gave the highest value at 26.77 wt%. In general, it can be seen that the first weight loss for amorphous carbon was low in the temperature range of 750–850 °C. It is noteworthy also that the second weight loss corresponding to CNTs was higher at a catalyst Ni composition of 10 wt% (15.78 wt%) compared to other Ni composition in the catalyst. Additionally, only one of the samples tested showed a third weight loss (not shown in Table 3) between 600–800 °C which was CQNs produced using Ni/CaO (15) at 800 °C with 4.534 wt% in the temperature range of 684–700 °C. This weight loss was presumably attributable to more stable carbon species such as graphitic soot [16,42].

### 3.7. Cyclic Voltammetry of Nanocomposite-Modified Screen-Printed Electrodes

**Figure 10:**
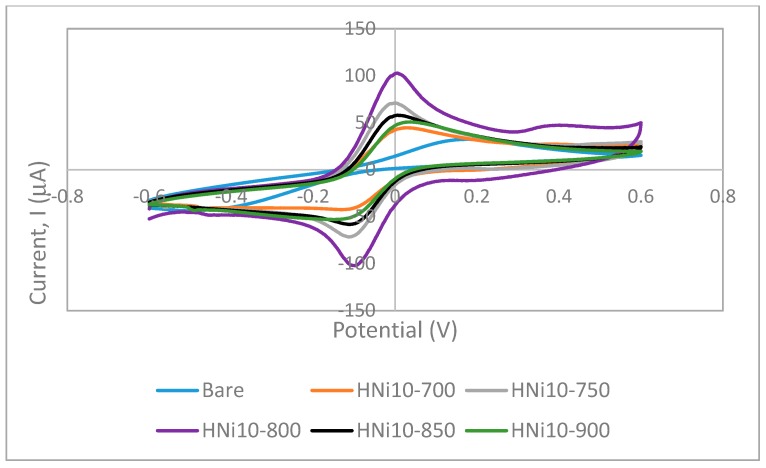
Cyclic voltammetry curves for bare screen printed carbon electrode, SPCEs and SPCEs modified using CQNs catalyzed using Ni/CaO (HNi) in 0.1 M KCl and 0.01 M K_3_[Fe(CN)_6_] at a scan rate of 100 mVs^-1^.

**Figure 11:**
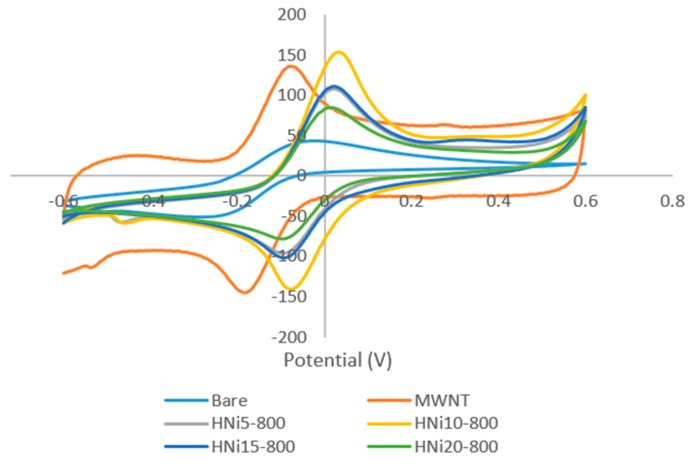
Cyclic voltammetry curves for bare SPCEs and SPCEs modified using CQNs catalyzed using Ni/CaO (HNi) compared with MWNT-modified SPCE in 0.1 M KCl and 0.01 M K_3_[Fe(CN)_6_] at a scan rate of 100 mVs^-1^.

The CV curves for the HNi10-modified SPCEs with varying CVD temperature are presented in Figure 10. While Figure 11 presents the CV curves for HNi-modified SPCEs at varying Ni catalyst compositions. The CV curves exhibited one anodic peak (upwards) and one cathodic peak (downwards), which indicated that the oxidation and reduction processes for ferrocyanide/ferricyanide redox couple had occurred on the electrode surface.

Figure 10 shows the CV curve for SPCEs modified using CQNs catalyzed by Ni/CaO (HNi), MWNT–COOH-modified SPCE, and bare SPCE. When the SPCEs were modified using Ni catalyzed CQNs (HNi) the CV response also had generally increased compared to bare SPCEs. Additionally, it can also be observed from Figure 10 that there was a slight shift of the CV peak to the right towards more positive potentials for the CV curve of Ni catalyzed CQNs compared to that of bare SPCEs and MWNT–COOH-modified SPCEs.

As the Ni content increased in CQNs, the average anodic peak current increased until Ni was at 10 wt%. When Ni content increased further, the CV response also decreased. However, only CQNs catalyzed with Ni/CaO (10 wt%) exhibited the higher CV response compared to commercial MWNT–COOH. Generally, the higher peak current for the Ni catalyzed CQNs was probably due to the existence of Ni in the nanocomposite, which influences its conductivity [43]. The average anodic peak current increases in correlation with the increment of the I_G_/I_D_ ratio (from Raman spectroscopy) of the material used for modification. The HNi10-800 with the highest average CV peak current also displayed the highest I_G_/I_D_ ratio of 1.30. The general trend observed was an increment in the peak current and I_G_/I_D_ ratio with increasing Ni content up to 10 wt% before a decrease in both values as the Ni content further increased. As mentioned earlier, the Raman I_G_/I_D_ ratio can be used as an indication of the electrochemical performance of the nanomaterials as there seems to be a good correlation of the ratio value with the anodic peak current recorded. It can be seen that the anodic peak current of HNi-modified SPCEs were generally better than bare SPCEs and MWNT–COOH-modified SPCEs. The high CV response could be attributed to the high graphitization of the CNTs in HNi10-800, which is favourable for electron transfer. A lower graphitization was observed for Ni content higher than 10 wt% since a high metal content in the catalyst could cause aggregation of the metal into larger metal clusters with weaker catalytic effects, therefore producing CNTs with larger diameters and poorer crystal structures compared to when lower catalyst metal concentrations are used [34].

**Figure 12:**
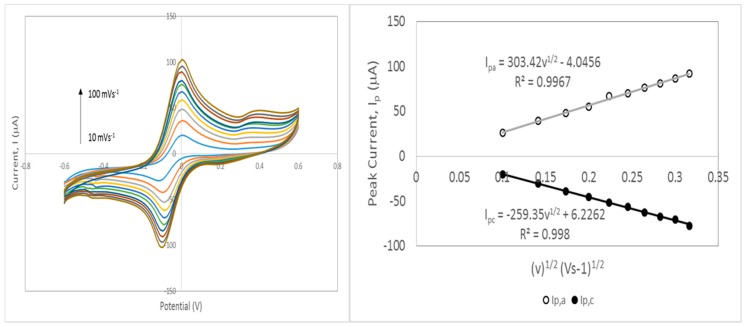
Cyclic voltammetry curves for scan rate studies of HNi10-800-modified SPCEs at potential scan rates of 10 to 100 mVs^−1^ and plots of the corresponding peak current versus the square root of the scan rate (√v) in 0.1 M KCl and 0.01M K_3_[Fe(CN)_6_]

Furthermore, the separation of peak potentials (ΔEp) were found to be lower for HNi-modified SPCEs (115–127 mV) compared to bare SPCEs indicating faster electron transfer. Meanwhile, ΔEp for HNi10-800-modified SPCEs was found to be only slightly higher than that of MWNT–COOH-modified SPCE (109 mV) indicating a comparable electron transfer rate. From the plot of CV peak current (i_p_) versus square root of the scan rate (√v) for HNi-modified SPCEs (Figure 12), the peak current gradually increased proportionally to √v which indicated a quasi-reversible surface electrode process. A higher slope (of i_p_ versus √v) with a linear trend indicates a higher electroactive surface area [44]. The linear variation of peak currents towards the square root of the scan rate also indicates that the mass transport of the species onto the electrode surface was controlled by diffusion [45].

#### 3.7.1. Electroactive Surface Area of HNi Nanocomposite-Modified Screen-Printed Electrode

In this work we used cyclic voltammetry and the Randles–Sevcik equation to determine the electroactive areas of the bare and modified SPCEs. The Randles–Sevcik equation is as follows:

(1) $$i_{p}=k\cdot n^{\frac{3}{2}}\cdot A\sqrt{Dv}\cdot C$$

where *k* is a constant of 2.69 × 10^5^ (Cmol^−1^v^−1/2^), *n* is the number of electrons appearing in the half reaction of the redox couple, *A* is the electrode area (cm^2^), *D* is the analyte diffusion coefficient (cm^2^s^−1^), *C* is the analyte concentration (mol), and *v* is the potential scan rate (Vs^−1^). In this work, *v* was measured from 10 to 100 mVs^−1^. When the peak current, i_p_ was plotted against the square root of the scan rate (√*v*), the slope (i_p_/√*v*) was given by:

(2) $$slope=\frac{i_{p}}{\sqrt{v}}=k\cdot n^{\frac{3}{2}}\cdot A\sqrt{D}\cdot C$$

Rearranging Equation (2) enables the determination of the electrode area, A:

(3) $$A=\frac{\frac{i_{p}}{\sqrt{v}}}{k\cdot n^{\frac{3}{2}}\cdot \sqrt{D}\cdot C}$$

Therefore, Equation (3) was used to determine the electroactive area, A.

The representative CV curves and plot of i_p_ versus (√v) for HNi10-modified SPCEs is presented in Figure 12.

From Table 3, HNi10-800-modified SPCEs gave the highest electroactive surface area at 0.380 cm^2^ compared to that of bare electrode at 0.098 cm^2^, which was more than a 5 fold increase compared to the geometrical area. As the CVD temperature increased, the surface area of HNi-modified SPCEs increased until 800 °C. Compared to the geometrical area (0.0707 cm^2^) and area of MWNT–COOH-modified SPCEs (A = 0.299 cm^2^), HNi10-750, HNi10-800, and HNi10-850 gave higher electroactive surface area values, which may be attributed to higher graphitization of the samples. Furthermore, these results imply that using CQNs catalyzed by Ni is a good alternative for MWNT–COOH substitution in electrode modification because the CQN displayed comparable electrochemical responses even if it contained a smaller amount of CNTs compared to the commercial MWNT–COOH.

#### 3.7.2. Estimation of Kinetic Parameter: Heterogeneous Electron Transfer Rate Constant (*k_0_*)

Additionally, the CV analysis can also be used to determine the heterogeneous electron transfer rate constant (*k_0_*) of the electrode surface. The rate constant indicates the speed of electron transfer between an electroactive species and an electrode surface. A common method of determining *k_0_* value is by using the Nicholson method which relates the dimensionless kinetic parameter *ψ* (psi) to the heterogeneous electron transfer rate *k_0_* with the following equation.

(4) $$\psi =k_{0}{\left[\pi Dn\nu F/\left(RT\right)\right]}^{-\frac{1}{2}}$$

where *D* is the diffusion coefficient, *n* is the number of electrons, *F* is the Faraday constant, *R* is the gas constant, and *T* is temperature (K). Subsequently, the *ψ* (psi) value is related to ΔEp via a table or working curve (*n*∆Ep versus log *ψ* plot for each scan rate *v*) provided by Nicholson [46,47]. Lavagnini [45] then revised the Nicholson method and proposed the following equation which relates *ψ* and values ΔEp for each scan rate.

(5) $$\psi =\left(-0.6288+0.0021\Delta E_{p}\right)/\left(1-0.017\Delta E_{p}\right)$$

Subsequently, the *k_0_* value is the slope of the plot of *ψ* versus [π*DnvF/(RT)*]^−1/2^ or *ψ* versus 32.79v^−1/2^ in this work, where the 32.79 factor was obtained by substituting the [π*DnF/(RT)*]^−1/2^ (from Equation (4)) with the following values: *D* = 7.6 × 10^−6^ cm^2^s^−1^, *F* = 96,485 C mol^−1^, *R* = 8.314 JK^−1^mol^−1^, and *T*= 298.15 K [45]. The HNi10-800-modified SPCEs were analyzed via CV and the heterogeneous electron transfer rate constant, *k_0_* was determined to be 1.1 × 10^−2^ cms^−1^ which is higher than the *k_0_* values for glassy carbon electrode (GCE) and carbon paste electrode (CPE) which were reported as 1.02 × 10^−3^ cms^−1^ and 3.6 × 10^−4^ cms^−1^, respectively [45,48].

## 4. Conclusions

The XRD results showed a temperature of at least 700 °C was needed for the formation of CNTs in its CQN nanocomposites. Scanning electron microscopy images also showed the presence of CNTs, CaO, and catalysts. Thermogravimetric analysis showed that the synthesized CQNs started to decompose at around 300 °C, which was due to the amorphous carbon and a second weight loss at 400 and 600 °C was also observed, which was due to the presence of CNTs. Raman analysis showed that 700–800 °C was a suitable temperature range to obtain well graphitized CQNs using Ni catalysts supported on CaO, obtained from carbonate stones. Raman spectroscopy indicated that the catalyst composition for highest I_G_/I_D_ ratio was Ni/CaO (10 wt%) synthesized at 800 °C. As screen-printed electrode modifiers, all of the CQN samples studied in this work showed increased CV response compared to bare SPCEs with HNi10-800 giving the highest peak current value and it is in parallel with the Raman spectroscopy results. The electroactive surface area of the modified SPCEs were the highest when HNi10-800 was used as the electrode modifier which was also probably due to the high graphitization of the material compared to other samples tested. From the CV, it could also be seen that HNi10-800-modified SPCEs gave faster electron transfers than bare SPCEs and this was possibly a better SPCE modifier than MWNT–COOH as it displayed higher CV peak currents and an electron transfer that was comparable.

## Figures and Tables

**Table 1:** Surface area and porosity of CQNs * synthesized using Ni/CaO.

No.	Sample	Catalyst Ni (wt%)	BET * Surface Area (m^2^/g)	Total Pore Volume (cm^3^/g)	BJH * Average Pore Diameter(Å)
1	HNi10-700	10	13	0.084	260
2	HNi10-750	10	12	0.078	250
3	HNi10-800	10	10	0.069	290
4	HNi10-850	10	6	0.051	340
5	HNi10-900	10	7	0.063	360
6	HNi5-800	5	8	0.067	330
7	HNi15-800	15	11	0.069	240
8	HNi20-800	20	8	0.070	370

* CQNs: carbon nanotube-quicklime nanocomposites. * BET: Brunauer-Emmett-Teller. * BJH: Barret-Joyner-Halenda.

**Table 2:** Sample weight loss during TGA analysis.

Sample	Weight Loss 1		Weight Loss 2	
	T (°C)	wt%	T (°C)	wt%
HNi10-700	350–373	13.11	537–576	14.06
HNi10-750	330–423	2.73	537–655	15.26
HNi10-800	200–477	18.35	573–708	15.78
HNi10-850	300–452	2.98	540–600	11.47
HNi10-900	357–381	13.90	549–609	26.77
HNi5-800	345–369	17.24	537–579	8.68
HNi15-800	349–376	8.43	540–581	5.76
HNi20-800	342–367	13.46	523–568	8.83

**Table 3:** The Electroactive area of SPCEs modified using Ni/CaO-catalyzed CQNs (HNi).

Electrode	Geometrical Area (cm^2^)	Electroactive Area (cm^2^)	Roughness Factor
Bare	0.0707	0.098 ± 0.010	1.39
HNi10-700	0.0707	0.168 ± 0.031	2.37
HNi10-750	0.0707	0.334 ± 0.053	4.73
HNi10-800	0.0707	0.380 ± 0.051	5.37
HNi10-850	0.0707	0.277 ± 0.050	3.93
HNi10-900	0.0707	0.188 ± 0.026	2.66
